# Editorial: Are Rodent Models Fit for Investigation of Human Obesity and Related Diseases?

**DOI:** 10.3389/fnut.2017.00058

**Published:** 2017-12-01

**Authors:** Patrick C. Even, Sam Virtue, Nicholas M. Morton, Gilles Fromentin, Robert K. Semple

**Affiliations:** ^1^UMR Physiologie de la Nutrition et du Comportement Alimentaire, AgroParisTech, INRA, Université Paris Saclay, Paris, France; ^2^Metabolic Research Laboratories, Wellcome Trust-MRC Institute of Metabolic Science, University of Cambridge, Cambridge, United Kingdom; ^3^University/BHF Centre for Cardiovascular Sciences, Queens Medical Research Institute, University of Edinburgh, Edinburgh, United Kingdom

**Keywords:** obesity, disease, rodent models, non-alcoholic fatty liver disease, type 2 diabetes mellitus

**Editorial on the Research Topic**

**Are Rodent Models Fit for Investigation of Human Obesity and Related Diseases?**

Mice and rats provide the primary model systems in which the pathophysiology of obesity and its associated pandemic diseases are investigated. Rodent models have been instrumental in the garnering of numerous insights into fundamental pathophysiological mechanisms that are conserved across species. Nevertheless, in key respects rodent physiology is distinct from that of humans, and uncritical overreliance on rodent findings risks impeding translational progress toward improving human health. This research topic aimed to assess whether current use of mouse and rat models is appropriate to maximize generalizable insights and to minimize erroneous conclusions being drawn about human disease. It solicited 16 publications, encompassing 14 opinions or reviews and 2 original research articles, describing or evaluating many aspects of current research performed using rats or mice, covering both causes and consequences of obesity.

Type 2 diabetes is the main focus of three articles. Santosa et al. discuss the use of the rodent pancreatic beta cell to understand the signaling pathways involved in human beta cell differentiation. Laber and Cox and Thomsen et al. consider the successes for rodent modeling of human genome wide association studies for adiposity and type 2 diabetes mellitus (T2DM), encompassing similarities and differences between mouse and human genomes. They consider how animal models can be developed as more precise disease models of T2DM by targeted gene manipulation in the correct developmental and tissue context. Significant limitations of this strategy are also discussed.

Rodent modeling of two major obesity associated diseases, namely non-alcoholic fatty liver disease (NAFLD) and polycystic ovary syndrome (PCOS), is the focus of two articles. The use of monogenic mouse models to interrogate the pathophysiology and genetic predisposition to NAFLD is discussed by Mann et al. This points to interspecies differences and variability in experimental protocols as a limit for the extent to which results from rodent models can currently be extrapolated to humans. Huang-Doran and Franks reviewed the characteristics of the PCOS and assessed the adequacy of rodent models for investigation of this complex pathology. They highlight the variable recapitulation of the PCOS phenotype in rodents, and the relative lack of insulin resistance-related PCOS, quite unlike humans.

Rat models have been used for three decades to study facets of nutrition, endocrinology, the metabolic syndrome, obesity, lipid metabolism, vascular myocardial pathophysiology and pharmacology, and are the primary focus of several articles in the topic. Chusyd et al. review the physiological properties and metabolic profiles of rodent white adipose fat pads and compare these to white adipose depots in humans. The authors also elaborate on sexual dimorphism in adipose tissue depots and attempt to explain why premenopausal women generally have a healthier metabolic risk profile than men. Diane et al. discuss how the JCR:LA-cp rat has contributed to understanding of the gut’s contribution to increased production of chylomicron particles, and how ruminant-derived trans fatty acids participate in regulating lipid metabolism. The authors also consider modeling of PCOS in this strain of rat. Bi and Moran focused on the insights into the neural basis of food intake and body weight control that have been yielded by studies of the obese OLEF-T rat, a CCK-1 receptor knockout model. The OLEF-T rat has also been valuable in dissection of interactions among exercise and food intake, and in investigating the role of the DMH in energy balance.

Giles et al. report lessons learned from study of the OP/OR rat, including the observation that OP rats have disturbances in hypothalamic signaling pathways involved in energy homeostasis before obesity develops, and while still on a low-fat diet. They also highlight the sexual dimorphism of obesity and point out that this is often ignored because most preclinical studies are performed on male rodent models. The authors have extended the study of the obese rat model to address the menopause and breast cancer using surgical ovariectomy in OP/OR female rats to mimic loss of ovarian function. Surgical ovariectomy was also used in mice by Chalvon-Demersay et al. to address the consequences of estrogen deficiency, however they suggest that differences observed even between rats and mice imply that extrapolations to humans must be made with caution. In the same article, the authors also discuss the “protein leverage hypothesis,” which proposes that insufficient protein intake may be a key factor in obesity development. The authors discuss in detail mechanisms whereby dietary protein levels may affect food intake.

The brain is the focus of two further articles. Poon and Leibowitz discuss the various techniques used for the administration of substances to rodents in studies of the neuronal and molecular mechanisms determining the behavioral outcomes of gestational exposure to non-illicit substances of abuse, such as excessive dietary fat, ethanol, and nicotine. Münzberg et al. suggest that environmental factors and genetic predisposition, rather than personal choices, are at the root of the obesity pandemic, and critically evaluate how rodent models can help to understand the contribution of hedonic neural processes to body weight regulation. They summarize how these models helped to solidify the new view that homeostatic and hedonic controls are closely interrelated, often acting in unison at the unconscious level to affect biologically adaptive responses.

Lutz and Bueter review the advantages and limitations of studying mechanisms underlying the benefits of bariatric surgical approaches to obesity treatment (Roux-en-Y gastric bypass and vertical sleeve gastrectomy) in rats and mice. They conclude that most animal models recapitulate remarkably well findings in humans, but indicate that animals larger than rats and mice may offer additional specific advantages.

Thornton reviews experiments in favor of the specific hypothesis that increased hydration leads to body weight and fat loss through a decrease in feeding. The hypothesis derives from a broad association between chronic dehydration, raised levels of angiotensin II and chronic diseases such as obesity, diabetes, cancer, and cardiovascular diseases. Proposed mechanisms involve an increase in metabolism due to expansion of cell volume by hydration.

Two original studies were published in this topic. Even and Blais sound a cautionary methodological note by demonstrating that the cost of thermoregulation in mice housed at room temperature strongly affects attempts to estimate thermogenic responses to feeding accurately, due to heat transfer between diet-induced thermogenesis and non-shivering thermogenesis. They suggest that these observations undermine the use of mice housed below thermoneutrality to model human disorders of energy balance. Finally, Lombardo et al. show that use of a simple standard diet, without additive agents and without caloric restriction, is sufficient to rescue high-fat feeding-induced insulin resistance and prevent the evolution of diabetes without the need for a hypocaloric diet.

The articles in this topic offer a cross section of current approaches to studying obesity and its associated diseases in rodents. They highlight successes and failures, as well as illustrating important methodological issues and some emerging hypotheses in the field. Collectively, they attest to the enduring utility of highly tractable rodent models of complex human metabolic disease and to the importance of continued and careful adjustment of rodents to the disease being modeled. They further raise awareness of areas where species-specific pathophysiology appears irrevocably distinct. Appropriately critical use of rodent models will remain vital to interrogation of pandemic human metabolic disease.

## Author Contributions

All authors have made a substantial, direct and intellectual contribution to the work, and approved it for publication.

## Conflict of Interest Statement

The authors declare that the research was conducted in the absence of any commercial or financial relationships that could be construed as a potential conflict of interest.

